# Reduced D2/D3 Receptor Binding of Extrastriatal and Striatal Regions in Temporal Lobe Epilepsy

**DOI:** 10.1371/journal.pone.0141098

**Published:** 2015-11-06

**Authors:** Viviane E. Bernedo Paredes, Hans-Georg Buchholz, Martin Gartenschläger, Markus Breimhorst, Mathias Schreckenberger, Konrad J. Werhahn

**Affiliations:** 1 Department of Neurology, University Medical Center of the Johannes Gutenberg University, Mainz, Germany; 2 Department of Nuclear Medicine, University Medical Center of the Johannes Gutenberg University, Mainz, Germany; Chiba University Center for Forensic Mental Health, JAPAN

## Abstract

**Objective:**

Dopamine is an endogenous neuromodulator in cortical circuits and the basal ganglia. In animal models of temporal lobe epilepsy (TLE), seizure threshold is modulated to some extent by dopamine, with D1-receptors having a pro- and D2-receptors an anticonvulsant effect. We aimed to extend our previously reported results on decreased D2/D3 receptor binding in the lateral epileptogenic temporal lobe and to correlate them with demographic and seizure variables to gain a more comprehensive understanding of the underlying involvement of the dopaminergic system in the epileptogenesis of TLE.

**Methods:**

To quantify D2/D3 receptor binding, we studied 21 patients with TLE and hippocampal sclerosis (13 left- and eight right-sided) and 18 controls using PET with the high-affinity dopamine D2/D3-receptor ligand ^18^F-Fallypride to image striatal and extrastriatal binding. TLE was defined by interictal and ictal video-EEG, MRI and ^18^F-Fluorodeoxyglucose PET. Voxel-based statistical and regions-of-interest analyses were performed.

**Results:**

^18^F-Fallypride binding potential was significantly reduced in the affected temporal lobe and bilateral putamen. A positive correlation between age at onset of epilepsy and [^18^F]FP BP_nd_ (binding potential non-displaceable) in temporal regions on the epileptogenic side was found, as well as a negative correlation between epilepsy duration and [^18^F]FP BP_nd_ in the temporal pole on the epileptogenic side and a positive correlation between the estimated number of lifetime GTCS and [^18^F]FP BP_nd_ in the hippocampus on the epileptogenic side.

**Significance:**

The areas of reduced D2/D3 receptor availability correspond to “the irritative zone” surrounding the epileptogenic area. Moreover, reduced D2/D3 receptor availability was detectable in the basal ganglia, which are suspected to be involved in a control circuit for epileptic seizures. The correlational analysis additionally suggests that increased epilepsy duration leads to increasing impairment of the dopaminergic system.

## Introduction

The dopaminergic system in epilepsy has received much interest, especially in imaging and genetic studies, which have confirmed the role of dopamine-mediated neurotransmission (for review see Ref. [1]). A reduction of striatal dopamine uptake in [^18^F]-fluoro-L-Dopa-PET has been described in patients with ring chromosome 20 mosaicism epilepsy [2] and in drug-resistant focal and generalized epilepsies [3, 4]. Furthermore, reduced dopamine transporter binding has been reported for juvenile myoclonic epilepsy in the substantia nigra and ventral tegmentum [5, 6] and epilepsy with tonic-clonic seizures in the putamen [7], suggesting that dopaminergic alterations may be related to the pronounced motor manifestation of syndrome-related seizures. Further studies have focused on the role of the dopaminergic system within the basal ganglia-thalamocortical circuitry and its assumed ability to control the propagation of seizures [8].

Alterations of D1- and D2-like receptors have been shown in different epilepsy syndromes. Reduced striatal D1-receptor binding has been shown in autosomal dominant nocturnal frontal lobe epilepsy [9]. D2/D3-receptor binding was reduced in the bilateral posterior putamen in patients with juvenile myoclonic epilepsy [10], while in a limited number of patients with mesial temporal lobe epilepsy (TLE) and hippocampal sclerosis reduced D2/D3-receptor binding was only present adjacent to the epileptogenic focus [11], indicating an alteration of the extrastriatal dopaminergic system.

In this study, we collected PET data for patients with TLE and hippocampal sclerosis and controls, employing the D2/D3 receptor ligand ^18^F-Fallypride to image striatal and extrastriatal D2/D3-receptor binding, and then performed voxel-based and regions-of-interest analyses on this data. We aimed to quantify D2/D3-receptor binding in order to further elucidate the role of these receptors in the pathophysiology of TLE and potentially to identify clinical and demographic influencing factors.

## Materials and Methods

### Patients and controls

The study was approved by the local ethical committee (Medical Association Rhineland-Palatinate) and national radiation safety authority (Federal Office for Radiation Protection) and patients gave written and informed consent according to the Declaration of Helsinki. We studied 21 patients with TLE (n = 13/8 for left/right-sided temporal focus; mean age±SD, 38.9±2.9 years; clinical and demographic details in Tables 1 and 2) and 18 healthy male controls (mean age, 30.4±4.6 years; non-smokers; no history of drug abuse; no history or evidence of neurological or psychiatric disorders; no women were included for ethical reasons; normal structural MRIs; all right-handed). Four patients were included in our previous study [11]. Patient inclusion criteria were: (i) clinically documented history of drug-resistant mesial TLE; (ii) scalp video-EEG recording of at least two spontaneous seizures with ictal EEG seizure pattern from temporal regions; (iii) brain MRI performed no later than six months before or after PET investigations; and (iv) standard MRI criteria for hippocampal sclerosis (hippocampal atrophy, increased T2 signal and loss of internal hippocampal architecture).

**Table 1:** Demographic and clinical features of patients.

Demographic features		TLE^a^ all	TLE left	TLE right
Sex	male/female	9/12	5/8	4/4
Lateralization	left/right	13/8	13/0	0/8
Handedness	left/right	3^b^/18	1^b^/12	2/6
Nicotine	yes/no	4/17	2/11	2/6
**Clinical features**				
Initial precipitating incidents				
*Febrile convulsions*	yes/no/unknown	8/9/4	5/6/2	3/3/2
*Head injury*	yes/no	2/19	2/11	0/8
Epilepsy onset^c^	age (years)	11.5±9.0	11.8±9.5	11.1±8.8
Epilepsy duration^c^	years	27.6±16.5	23.2±13.9	34.6±18.9
Aura	yes/no	18/3	11/2	7/1
Complex partial seizures	yes/no	21/0	13/0	8/0
Secondarily generalized seizures	yes/no	16/5	11/2	5/3
Ictal dystonia	yes/no	8/13	5/8	3/5
Ictal version	yes/no	5/16	3/10	2/6

^a^ TLE = temporal lobe epilepsy

^b^ one patient was relearned from left to right

^c^ Clinical and demographic data did not differ between right and left TLE patients.

**Table 2:** Specific clinical characteristics of patients.

Patient No.	Ictal EEG	Interictal EEG	FDG PET	AED therapy	Surgery	Engel’s class
1	L temp (n = 3)	100% L temp	Y	LEV LTG	Y	Ia
2	L temp (n = 3)	52% L temp 48% R temp	Y	GBP LEV VPA	Y	Ib
3	L temp (n = 2)	100% L temp	Y	OXC LEV	Y	Ia
4	L temp (n = 7)	75% L temp 25% R temp	Y	CBZ LEV	Y	Ia
5	L temp (n = 3)	100% L temp	N	LEV TPM	Y	Ia
6	R temp (n = 2)	100% R temp	Y	LEV LTG	Y	Ib
7	R temp (n = 3)	95% R temp 5% L temp	Y	LEV LCM	Y	Ia
8	L temp (n = 4)	100% L temp	Y	LEV LTG	Y	Ia
9	L temp (n = 13)	98% L temp 2% R temp	Y	LEV	Y	Ia
10	R temp (n = 6)	100% R temp	Y	LCM	N	-
11	L temp (n = 2)	20% L temp 80% R temp	Y	LEV PGB	N	-
12	R temp (n = 6)	100% R temp	Y	LEV LCM	Y	Ia
13	R temp (n = 8)	73% R temp 24% L temp	Y	LEV LTG	Y	Ia
14	L temp (n = 3)	100% L temp	Y	LEV LCM	Y	Ia
15	L temp (n = 5)	47% L temp 53% R temp	Y	LTG TPM	Y	Ic
16	R temp (n = 4)	94% R temp 6% L temp	Y	LEV LCM	N	-
17	R temp (n = 2)	83% R temp 17% L temp	Y	LEV LTG	Y	Ia
18	L temp (n = 4)	100% L temp	Y	LEV	Y	Ia
19	L temp (n = 3)	100% L temp	Y	LEV PGB	Y	Id
20	R temp (n = 4)	100% R temp	Y	LEV LCM	Y	Ia
21	L temp (n = 8)	100% L temp	Y	LEV LCM	Y	Ia

FDG = Fluorodeoxyglucose, AED = Antiepileptic drugs, CBZ = Carbamazepine, GBP = Gabapentin, LCM = Lacosamide, LEV = Levetiracetam, LTG = Lamotrigine, OXC = Oxcarbazepine, PGB = Pregabalin, TPM = Topiramate, VPA = Valproic Acid, temp = temporal, L = Left, R = Right, Y = Yes, N = No.

### EEG methods

EEG recordings were carried out using 32-channel surface plus sphenoidal electrodes (in 15 of 21 patients) placed according to the international 10–20 system. One patient was also studied with subdural grid electrodes. Interictal and ictal EEG activity was recorded separately to the PET scan, and lateralization to the left or right temporal lobe was based on congruent interictal and ictal data with at least two typical seizures in each case.

### ^18^F-Fallypride PET

After a 10 min transmission scan, a 180 min dynamic emission recording in 3D mode consisting of 39 frames, increasing in duration from 20 to 600 s, was initiated with the ECAT EXACT tomograph [12] (Siemens/CTI, Knoxville, TN, U.S.A.) upon intravenous bolus injection of 189.0±25.2 MBq and 184.8±23.6 MBq [^18^F]FP for patients and controls, respectively.

PET images were reconstructed using filtered back projection and Hamming filter (width: 4 mm) and corrected for scatter and attenuation. All dynamic PET recordings were transferred to a Sun Workstation (Sun Microsystems) for further data analysis using MPI tool (ATV Erfstadt, Germany). Although great care was taken to minimize head movement by a head holder, displacement was noted in some cases. Correction was realized under the condition of no head movement during the first 10 min. The emission recording summed over this time interval was manually resliced in the anterior–posterior commissure orientation. All subsequent emission frames were realigned to the consecutively corrected summed recording to optimize matching of the cerebral contours. The quality of the final alignment was assessed framewise by visual inspection and striatal time–radioactivity curves were extracted and evaluated for movement artefacts. The final dynamic emission recordings were summed. Parametric images of binding potential were obtained using the simplified reference tissue model [13]. The free and non-specific ligand kinetic is based on the time-activity curve of a cerebellar reference region assumed to be devoid of D2-/D3-specific binding [14]. Individual transaxial MRIs were co-registered with the corrected early summed emission recordings and parametric images of binding potential in the anterior-posterior commissure and bihippocampal orientations.

#### Regions of interest

**Fig 1:**
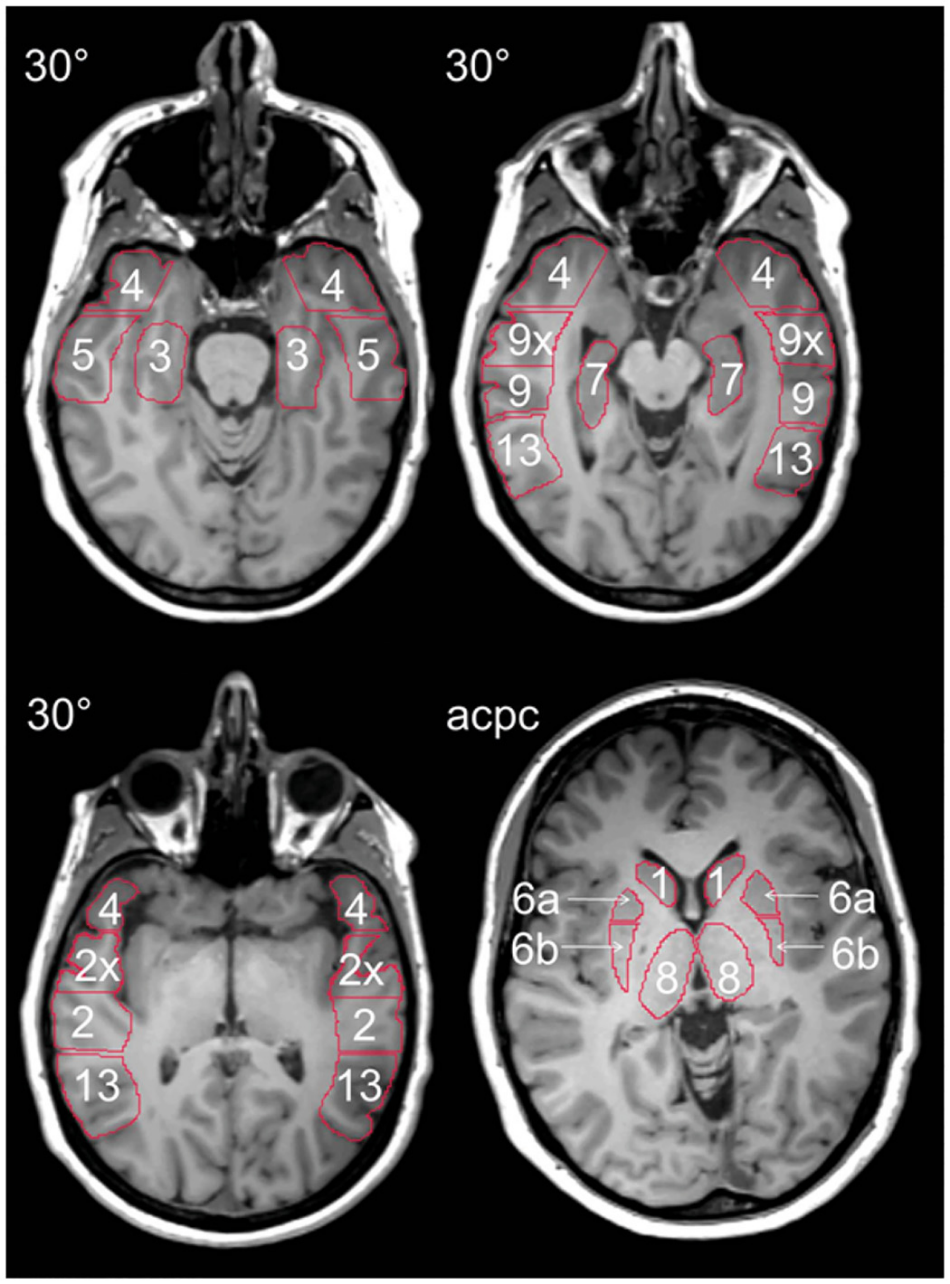
Regions of interest for PET data analysis. ROIs were outlined manually on patient and control MRIs. 1: caudate nucleus (caput); 2x: superior temporal gyrus (leading part); 2: superior temporal gyrus; 3: parahippocampal gyrus; 4: temporal pole; 5: inferior temporal gyrus; 6: putamen (a = anterior, b = posterior); 7: hippocampus; 8: thalamus; 9x: middle temporal gyrus (leading part); 9: middle temporal gyrus; 13: inferior parietal lobule. Not shown: midbrain and similar ROIs just drawn in consecutive planes. 30°: bihippocampal orientation; acpc: anterior-posterior commissure orientation.

50 hypothesis-controlled ROIs were drawn on the registered individual MRI and copied to the parametric binding potential images. Following Merlet et al. [15], ROIs were outlined anatomically following the grey matter ribbon. In the anterior-posterior commissure orientation, bilateral ROIs were drawn in the caudate nucleus (caput), anterior and posterior part of the putamen, thalamus (each drawn in three consecutive planes), and midbrain; and in the bihippocampal orientation, in the superior and middle temporal gyrus (as well as in their isolated leading parts), inferior temporal gyrus, temporal pole (in three consecutive planes), inferior parietal lobule (in two planes), parahippocampal gyrus and hippocampus. Calculated BP_nd_ values for ROIs drawn in consecutive planes were averaged (Fig 1), yielding a total of 14 ROIs on each side. Due to the scanner-specific average axial resolution of 5.0 mm (center) to 8.1 mm (at a distance of 20 cm to the center; R = 20 cm) full width at half-maximum [12], no ROI was drawn separately in the substantia nigra, though it plays a pivotal role in the dopaminergic system.

To detect intraindividual differences in local receptor concentrations, the variation of BP_nd_ for each ROI between affected and unaffected hemispheres was calculated by:

$$\Delta BP=\;\frac{BP_{epileptogenic}-\;BP_{unaffected}}{BP_{unaffected}}\times 100$$

#### Voxel-wise statistical analysis

Voxel-wise statistical analysis was performed with SPM8 (http://www.fil.ion.ucl.ac.uk/spm/software/spm8/). Integral images of the dynamics were spatially normalized using a ligand-specific template from ^18^F-Fallypride scans of healthy controls. The applied ^18^F-Fallypride template image for stereotactic normalization was taken from previous work [16]. Non-linear warping parameters were applied to the co-registered BP_nd_ images. Integral images and BP_nd_ images of all right-sided TLE patients were flipped to the left side. Normalized BP_nd_ images were smoothed using a 12 × 12 × 12 mm^3^ full-width at half-maximum isotropic Gaussian kernel that accommodates interindividual anatomy variability [17].

### 18-fluoro-2-deoxyglucose (FDG)-PET

FDG-PET was performed in 20 of 21 patients as a routine presurgical investigation following the guidelines for brain imaging [18] with an average FDG dose of 255.8±69.1 MBq. The FDG-PET data was included in this report to correlate any changes in brain metabolism with ^18^F-Fallypride binding. Only data acquired on the same scanner as that used for the ^18^F-Fallypride-PET (16 patients; 11/5 with left-/right-sided TLE) were used for the analysis. Image reconstruction was performed using filtered back projection and a Hamming filter. If no relevant head movement was observed, three 10-min frames were combined to a single image. FDG-PET was analyzed using NEUROSTAT and 3D standard surface projections.

FDG-PETs were not only visually analyzed for focal hypometabolism but also co-registered with parametric images of BP_nd_ in the anterior-posterior commissure and bihippocampal orientations. ROIs were co-registered in the individual parametric images of BP_nd_ and FDG-PET to check for a causal relationship between glucose metabolism and dopamine receptor concentration. The standardized uptake value (SUV) was calculated as the ratio of the radioactivity concentration and the administered activity divided by the body weight.

### Statistical analysis

**Fig 2:**
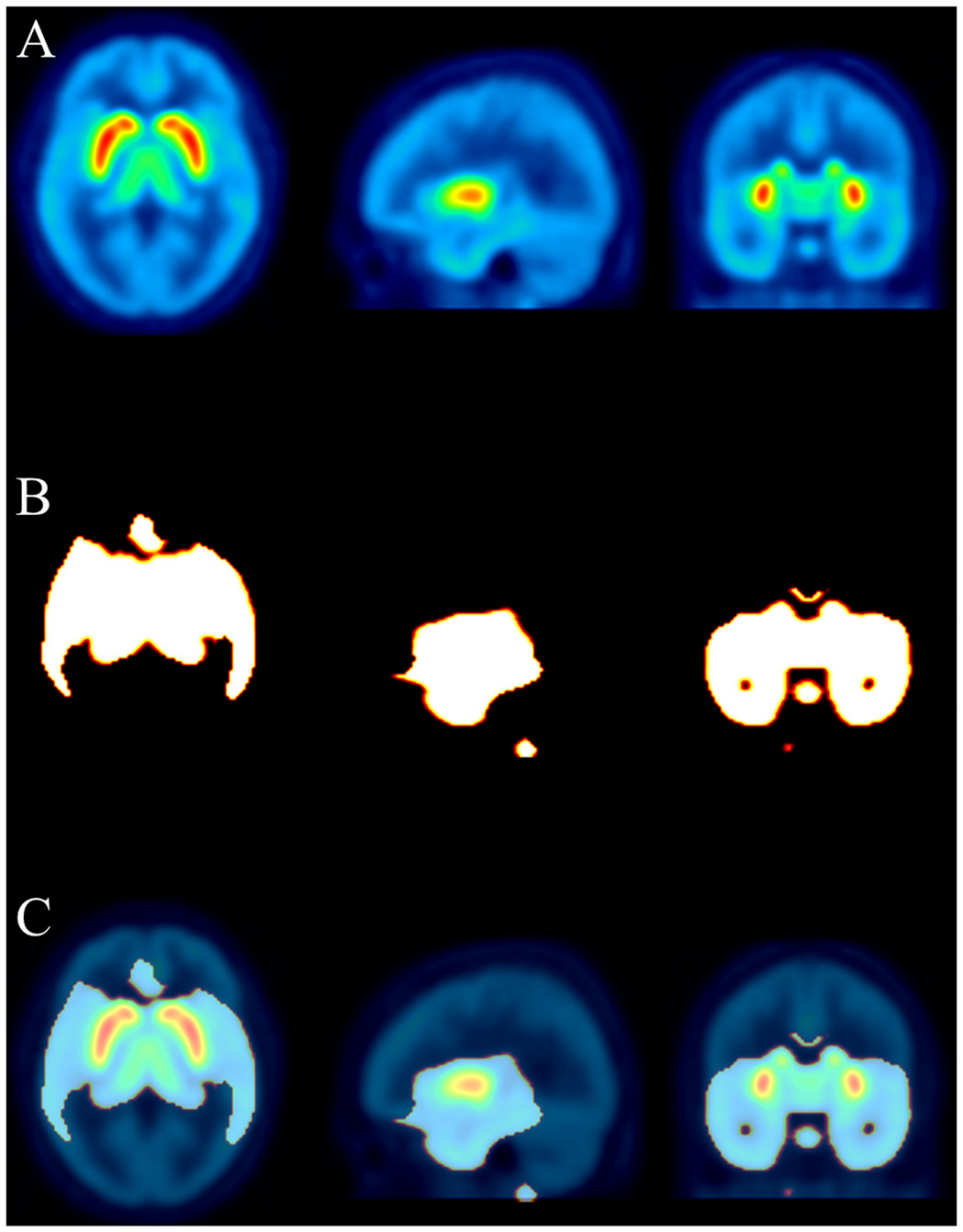
Binary mask for small volume correction. ^18^F-Fallypride template image for stereotactic normalization (A). Binary mask for small volume correction consisting of all hypotheses-controlled regions of interst equivalent to BP_nd_ >0.35 (B) and overlay of ^18^F-Fallypride template image and binary mask (C).

For group comparisons, we performed two-sample t-tests between the TLE patients and the healthy male controls on voxel-wise basis using SPM8. Statistical parametric maps of positive and negative contrasts were calculated using a threshold of p<0.0001 uncorrected (equates to p<0.05 corrected at cluster level) and a minimal cluster size >20 voxels. As implemented in SPM8, one-way analyses of covariance were performed by entering the global BP_nd_ (mean of all BP_nd_ values within the brain mask in order to eliminate any unspecific ^18^F-Fallypride enrichment, e.g., in the mucus membranes) as a covariate. Additionally, in order to correct for multiple comparison, we used a small volume correction implemented in the SPM8 software. Therefore, we applied a binary mask (Fig 2) including all hypothesized-controlled selected regions already defined for ROI analysis. Coordinates of significant clusters were reported using MNI coordinates.

For ROI analyses (in the whole group as well as in the left- and right-side affected patients), we tested possible differences between the epileptogenic and non-epileptogenic side in [^18^F]FP BP_nd_ and [^18^F]FDG SUV for each ROI using paired t-tests. ROI [^18^F]FP BP_nd_ values between patients and controls were compared by one-way analyses of covariance, defining “group” (patients vs. controls) as a between-subject factor and global BP_nd_ as a covariate with potential influence on [^18^F]FP BP_nd_. ROI [^18^F]FP BP_nd_ values between patients with left-sided and those with right-sided TLE were compared by one-way analyses of covariance, defining “group” (patients with left-sided vs. patients with right-sided TLE) as a between-subject factor and global BP_nd_ as a covariate with potential influence on [^18^F]FP BP_nd_. ROI-based linear relationships between regional [^18^F]FP BP_nd_ and age at onset, epilepsy duration, estimated number of lifetime generalized tonic-clonic seizures (GTCS), seizure frequency and spike frequency on the epileptogenic and non-epileptogenic sides were tested using partial correlation coefficient (two-sided) controlling for global BP_nd_. Normality of demographic/clinical data was checked using the Kolmogorov-Smirnov test. If necessary, data were log-transformed (estimated number of lifetime GTCS, seizure frequency and spike frequency) after adding a small constant to avoid zero values. Significant results were defined as p<0.05. All values are reported as mean±standard deviation (SD). All calculations were performed using SPSS 17.0 (SPSS Inc. Chicago, IL, U.S.A.).

## Results

### Regional uptake of [^18^F]Fallypride

**Fig 3:**
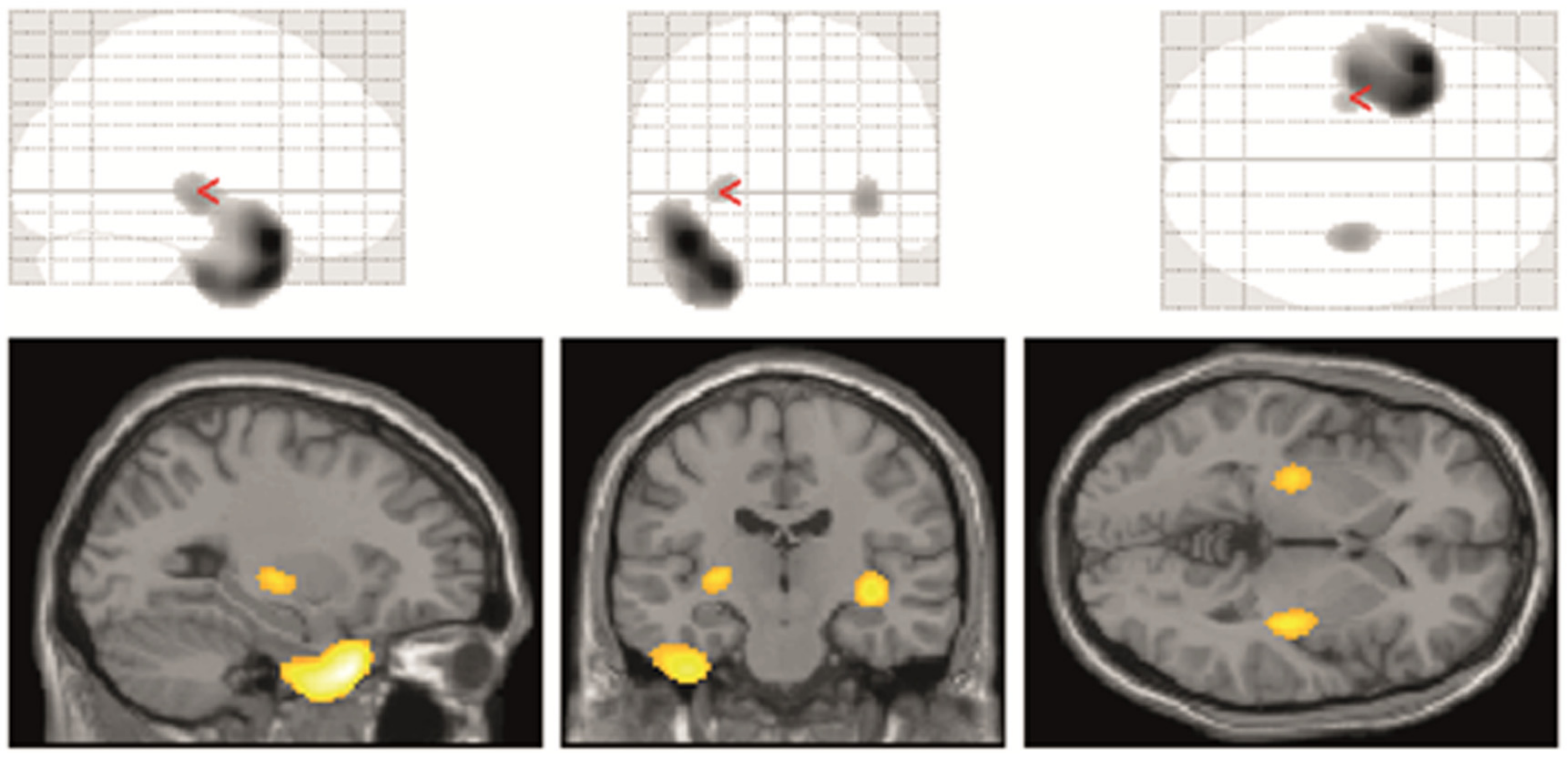
Statistical parametric mapping results. Statistical parametric mapping results (contrast controls–patients). Glass brain of the statistical map (top) and superposition of statistical maps onto an averaged MRI (bottom). The statistical threshold is p<0.0001 uncorrected (equates to p<0.05 corrected at cluster level). (Yellow color indicates decrease in [^18^F]FP BP_nd_ in patients).

The voxel-wise statistical analysis of the group comparison with controls revealed a significantly reduced [^18^F]FP BP_nd_ in the bilateral basal ganglia and the epileptogenic temporal lobe. As shown in Fig 3, [^18^F]FP BP_nd_ was decreased in the anterior (x = -28, y = 12, z = -38; z = 6.03) and lateral sections, particularly within the anterior field of the superior (x = -42, y = 14, z = -24; z = 6,14) and inferior (x = -36, y = -12, z = -42; z = 5.40) temporal gyrus of the epileptogenic temporal lobe. By contrast, mesial temporal areas did not show any significant [^18^F]FP BP_nd_ alteration. There was no significant [^18^F]FP BP_nd_ alteration seen in the unaffected temporal lobe or in the reverse contrast of patients versus controls. The decreased [^18^F]FP uptake in the basal ganglia was observed in the bilateral putamen (x = -26, y = -18, z = 2; z = 4.37 on the epileptogenic and x = 36, y = -18, z = -4; z = 4.69 on the non-epileptogenic side).

ROI-based comparison of the control and patient groups confirmed these results (S1 Table). [^18^F]FP BP_nd_ was significantly reduced in the temporal pole (0.79±0.15 vs. 0.44±0.15; p<0.001) and superior temporal gyrus (0.59±0.15 vs. 0.44±0.15; p = 0.007) as well as in the inferior temporal gyrus (0.64±0.18 vs. 0.43±0.18; p<0.001) and middle temporal gyrus (0.52±0.16 vs. 0.40±0.16; p = 0.03) on the epileptogenic side. There was a significant reduction in [^18^F]FP BP_nd_ in the bilateral putamen. No significant difference was detectable between the affected and unaffected sides. However, the decrease in [^18^F]FP BP_nd_ was more prominent in the putamen ipsilateral to the epileptogenic focus since it showed a global BP_nd_ decrease in the anterior (19.49±2.22 vs. 17.48±2.21; p = 0.010) and posterior (19.02±2.24 vs. 17.49±2.23; p = 0.05) parts, while the contralateral putamen showed a decrease in [^18^F]FP uptake only in the posterior part (19.63±2.19 vs. 17.66±2.18; p = 0.01).

**Fig 4:**
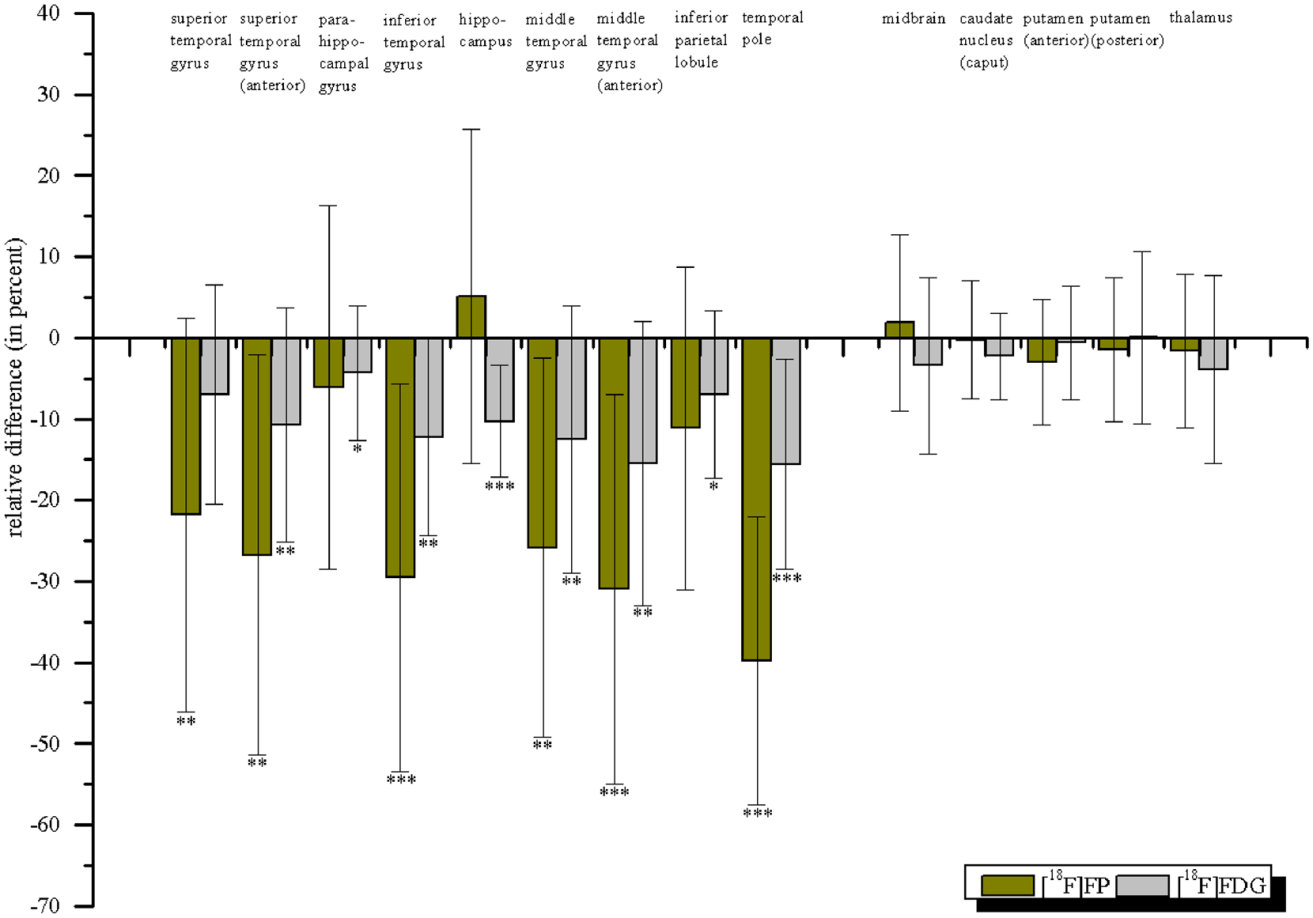
Mean relative [^18^F]FP binding potential and FDG uptake. Mean relative [^18^F]FP binding potential and FDG uptake in percent (±standard deviation) for patients with TLE (n = 16) given by ((BP_nd_ affected side—BP_nd_ unaffected side) / BP_nd_ unaffected side)*100 and (FDG uptake (SUV) affected side—FDG uptake (SUV) unaffected side) / FDG uptake (SUV) unaffected side)*100), respectively. Statistical differences refer to the comparison between affected and unaffected sides separately for each tracer (***p<0.001, **p<0.01, *p<0.05).

ROI-based intraindividual comparison of the epileptogenic and non-epileptogenic sides showed a more pronounced reduction of cortical [^18^F]FP binding on the epileptogenic side compared to the ROI-based quantification to the control group (Fig 4, S2 Table).

### [^18^F]Fallypride uptake according to the side of onset of epileptogenic activity

ROI-based comparison of left-sided and right-sided TLE patients showed an isolated and significant reduction in [^18^F]FP BP_nd_ in the superior temporal gyrus (0.63±0.20 vs. 0.28±0.22; p = 0.04) and anterior part of the superior temporal gyrus (0.69±0.22 vs. 0.31±0.22 p = 0.04) in patients with right-sided TLE. ROI-based intraindividual comparison of the epileptogenic and non-epileptogenic sides (data not shown) confirmed the results from the interindividual comparison. Moreover, relative to the non-epileptogenic side, patients with right-sided TLE exhibited a significant decrease in [^18^F]FP BP_nd_ in the thalamus on the epileptogenic side (1.69±0.43 vs. 1.93±0.44; p = 0.03), whereas in patients with left-sided TLE, there was a significant increase in [^18^F]FP BP_nd_ in the hippocampus on the epileptogenic side (0.94±0.21 vs. 0.84±0.20; p = 0.04).

### Regional uptake of [^18^F]Fluorodeoxyglucose

ROI-based intraindividual comparison of the epileptogenic and non-epileptogenic sides showed a decrease in [^18^F]FDG uptake (SUV), particularly in the hippocampus (3.79±0.94 vs. 4.22±1.02; p<0.001) and temporal pole (3.39±0.93 vs. 3.98±1.06; p<0.001), but also in the entire temporal lobe on the epileptogenic side, including the mesial and lateral aspects, with the exception of the superior temporal gyrus. In addition, the inferior parietal lobule (4.85±1.20 vs. 5.19±1.17; p = 0.01) dorsally following the temporal regions on the epileptogenic side showed a hypometabolism (Fig 4). No correlation was found between this hypometabolism and the decrease in [^18^F]FP BP_nd_.

ROI-based comparison of left- and right-sided TLE showed no significant alteration in [^18^F]FDG uptake (SUV).

### Correlation of [^18^F]FP BP_nd_ with clinical data

There was a positive correlation between the age at onset of epilepsy and [^18^F]FP BP_nd_, which involved solely temporal regions (inferior temporal gyrus: r = 0.46, p = 0.04; middle temporal gyrus anterior part: r = 0.50, p<0.03 and the temporal pole: r = 0.72, p<0.001) on the epileptogenic side. Furthermore, a negative correlation was detected between epilepsy duration and [^18^F]FP BP_nd_ in the temporal pole on the epileptogenic side (r = -0.39, p = 0.05). There was a positive correlation between the estimated number of lifetime GTCS and [^18^F]FP BP_nd_ in the hippocampus on the epileptogenic side (r = 0.47, p = 0.04). No correlation to [^18^F]FP BP_nd_ was observed for either seizure frequency or spike frequency.

## Discussion

Our study showed extensive reductions in D2 receptor availability *in vivo* in patients with TLE in brain areas ipsilateral to the epileptogenic focus consistent with previous preliminary findings [11]. In addition to confirming these findings in a larger and independent cohort, the study extends previous results, showing a decrease in [^18^F]Fallypride binding in extrastriatal and striatal brain areas afferent to the epileptogenic zone.

The detection of a bilateral decrease in [^18^F]FP BP_nd_ in the putamen is consistent with the presumed role of the dopaminergic system within the basal ganglia-thalamocortical circuitry, since the dorsal striatum (i.e., caudate and putamen) is considered a key structure in the control of epileptic seizure spread [8, 19–23]. This dopaminergic influence is thought to be differentially mediated by D1 and D2 dopamine receptors. Activation of the D2-mediated inhibitory pathway leads to an inhibition of the target areas within the thalamus and thereby to a reduced excitability of afferent cortical areas [24]. Assuming a high level of dopamine in epilepsy [25, 26] as a consequence of cortical hyperexcitability leading to a tonic release of dopamine [26], increased D2-mediated activation might serve to limit the spread of epileptic activity. This is in line with experimental data in rodents that show that pharmacological stimulation of D2-receptors in the striatum has a protective effect on pilocarpine-induced convulsions [21]. Results in patients with idiopathic generalized (genetic) epilepsy (juvenile myoclonic epilepsy) showing a decrease in [^18^F]FP BP_nd_ specifically in the bilateral posterior putamen [10] and also the detection of reduced [^18^F]FP BP_nd_ in the bilateral anterior caudate-putamen in pilocarpine-treated rats [27] seem to lend further support to the control of seizure spread through basal ganglia mechanisms. Our findings in patients with TLE might reflect increased activity of the glutamatergic corticofugal fibers due to cortical hyperexcitability leading to an increasing tonic release of dopamine and thereby downregulation of dopamine receptors [26].

If alterations of the dopaminergic system in patients with epilepsy are cause or consequence of seizures and epilepsy remains controversial and cannot be answered with the present study. However, arguments can be brought up for alterations being a consequence rather than the cause. In our study, a positive correlation between the age at onset of epilepsy and [^18^F]FP BP_nd_ was detected in the temporal lobe on the epileptogenic side. Furthermore, D2/D3-receptor binding negatively correlated with epilepsy duration in the temporal pole on the epileptogenic side. This is in line with the study of Rocha et al. [28] evaluating the expression and binding of dopamine receptors in cerebral cortex samples. The observation of unaffected D2-receptor protein expression in patients with TLE secondary to brain tumor or lesion is therefore explained by a longer duration of epilepsy and younger age at onset in patients with TLE due to hippocampal sclerosis. It was hypothesized that an increased duration of epilepsy and its assumed association with a progressive involvement of the adjacent cortical areas in the epileptogenic zone [28] leads to an increasing impairment of the dopaminergic system.

While dopamine D2 receptor availability was reduced in basal ganglia we found a relative increase of [^18^F]FP BP_nd_ in hippocampus ipsilateral to the epileptogenic focus pointing to a different pathophysiological role of D2-mediated dopaminergic influence in this area, which deserves discussion. There is evidence that the dopaminergic system plays a pivotal role in neurotoxicity and neuroprotection, with several studies indicating a D2 receptor-mediated neuroprotection (for review see Ref. [29]). With regard to further elucidating hippocampal cell death in epilepsy, the well characterized kainic acid model was used by Bozzi et al. [30] to show that in D2 receptor knock-out mice, but not in wild type mice, kainic acid-induced limbic seizures result in extensive hippocampal cell death by apoptosis. Interestingly, while Seidenberg et al. [31] showed that hippocampal volume decreases with increasing duration of epilepsy and moreover was negatively correlated to the estimated number of lifetime generalized seizures, our data show a significant increase in [^18^F]FP BP_nd_ in the hippocampus on the epileptogenic side compared to the non-epileptogenic side in patients with left-sided TLE. With regard to the assumed D2 receptor-mediated neuroprotection in the hippocampus, our results might be explained by an increased need for neuroprotection, and therefore, upregulation of D2 receptors in mesial temporal areas. This assumption is further supported by the observation of a positive correlation between the estimated number of lifetime GTCS (as a marker for severity of epilepsy) and [^18^F]FP BP_nd_ in the hippocampus on the epileptogenic side in TLE patients in our study. Additionally, several *in vivo* studies have shown that dopamine exerts an inhibitory influence on epileptogenesis in the hippocampus. Stereotactic microinjection studies with focal intrahippocampal injections of selective dopamine-receptor agonists and antagonists have shown this is due to dopamine D2 receptors (for review see Ref. [32]). Therefore, our results may also suggest a consecutive increase in dopamine D2-receptor availability in the hippocampus as a consequence of altered epileptic discharges and thus be preventative against evolving epileptic discharges.

Whether an isolated significant intraindividual increase in [^18^F]FP BP_nd_ in the hippocampus on the epileptogenic side in left-sided TLE patients in our study is epileptic-onset-side dependent or due to the small sample volume requires further study with a larger cohort. Nevertheless, a quantitative volumetric comparison of left- and right-sided TLE patients in patients with unilateral TLE [31] showed more widespread and pronounced white-matter volume loss in patients with right-sided TLE and thus agrees with our results showing a significantly reduced [^18^F]FP BP_nd_ in the superior temporal and anterior part of the superior temporal gyrus in right-sided compared to left-sided TLE patients.

Our study has several limitations. Due to the method employed and the missing autoradiographic studies on resected tissue, the explanatory power of this study has limitations in terms of whether the decrease in dopamine D2/D3-receptor availability is due to dopamine-receptor downregulation or increased dopamine occupancy leading to competitive displacement of [^18^F]FP. Recently, Rocha et al. [28] investigated the protein expression and binding of both D1 and D2 receptors in neocortex samples from 12 surgically treated TLE patients due to HCS. As compared to six control samples, using autopsy material, elevated D1-receptor protein expression and binding and reduced D2-receptor protein expression was verifiable in the neocortex of TLE patients. These results support dopamine-receptor downregulation as the cause of decreased [^18^F]FP BP_nd_ shown here. Contradictorily, in the same study, D2-receptor binding in the neocortex, measured by autoradiography using [^3^H]Raclopride, was unaffected in TLE patients compared to controls. This discrepancy might be explained by the known difference in the affinities of [^18^F]Fallypride and [^3^H]Raclopride to D2/D3 receptors as [^3^H]Raclopride, as a so called moderate-affinity PET tracer, is rather unsuitable for detection of extrastriatal binding [14].

The possible relationship between the dopaminergic neurotransmission involvement and morphological changes in TLE—as another possible explanation for the decrease in dopamine D2/D3-receptor availability shown in our study—was addressed by Bouilleret et al. [4]. The presynaptic dopaminergic influx was studied using [^18^F]-fluoro-L-Dopa-PET, glucose metabolism by using [^18^F]FDG-PET and morphologic cerebral changes by using voxel-based morphometry. While reduced [^18^F]-fluoro-L-dopa uptake was seen in the bilateral caudate, putamen and in the substantia nigra, no metabolic alterations and only mild gray matter volume reduction were observed in striatal regions without any changes in the substantia nigra. The authors reason that these discrepancies are indicative of the basal ganglia dopaminergic neurotransmission involvement not being related to structural subcortical abnormalities. To exclude metabolic alterations as cause for the dopamine receptor alterations shown in our study, we also performed [^18^F]FDG-PET and, comparable to the results of Bouilleret et al. [4], no correlation was found between FDG hypometabolism and decrease in [^18^F]FP BP_nd_.

As another possible limitation it should be mentioned, that although healthy controls and patients did not statistically differ due to their age, there was a trend toward an older age in the patient group, while age has already been shown to have a negative correlation with D2-receptor availability [33, 34] and might therefore have influenced our results. Additionally, due to known influence of gender and smoking behavior on D2-receptor availability [35], the solely non-smoking male control group might also have affected the results. It must be mentioned that all of these studies solely considered the basal ganglia, so that there is no evidence for extrastriatal structures.

Perspectively, a longitudinal [^18^F]FP-PET study of patients before and after epilepsy surgery to evaluate the reversibility of alterations in the dopaminergic system might be illuminative. Furthermore, to bridge the gap between electrophysiological data in rodents and our imaging study in humans, it might be useful to investigate an animal model of mesial TLE with classic hippocampal sclerosis by [^18^F]FP [36] and autoradiography.

For clinical practice, [^18^F]FP-PET might provide added value in the presurgical lateralization of MRI-negative TLE, where the reported degree as well as localization of [^18^F]FDG-PET hypometabolism and therefore its lateralizing value in MRI-negative TLE varies widely [37, 38]. Therefore, the lack of evidence for a structural lesion goes along with an increased risk of seizure recurrence after surgery [39], so that a better localizational tool such as [^18^F]FP-PET might improve the postsurgical outcome and hence should be investigated in a further study.

In summary, the areas of reduced D2/D3-receptor availability correspond to “the irritative zone” surrounding the epileptogenic onset, as well as to the basal ganglia which are suspected to be involved in a control circuit for epileptic seizures. Correlational results additionally suggest that increased duration of epilepsy leads to increasing impairment of the dopaminergic system.

## Supporting Information

S1 Dataset [^18^F]FP BP_nd_ ROI original data of the patient group. ^18^F-Fallypride binding potential scores for regions of interest on epileptogenic and non-epileptogenic sides for all patients. The regions of interest are denoted as described in Fig 1.
(XLS)

S2 Dataset [^18^F]FP BP_nd_ ROI original data of the control group. ^18^F-Fallypride binding potential scores for bilateral regions of interest for all controls. The regions of interest are denoted as described in Fig 1.
(XLS)

S3 Dataset [^18^F]FDG and [^18^F]FDG SUV ROI original data of the patient group. ^18^F-Fluorodeoxyglucose scores and its standardized uptake value for regions of interest on epileptogenic and non-epileptogenic sides for all patients. The regions of interest are denoted as described in Fig 1.
(XLS)

S4 Dataset Clinical features for all patients. (XLS)

S1 Table [^18^F]FP BP_nd_ ROI-based comparison of the control and patient groups. ^18^F-Fallypride binding potential mean scores (± standard deviation) and statistics for regions of interest on epileptogenic and non-epileptogenic sides of all patients compared to controls.
(DOC)

S2 Table [^18^F]FP BP_nd_ ROI-based comparison of the epileptogenic and non-epileptogenic sides. ^18^F-Fallypride binding potential mean scores (± standard deviation) and statistics for regions of interest on epileptogenic side compared to non-epileptogenic side of all patients.
(DOC)
