# Supplementary material for: Reduced D2/D3 Receptor Binding of Extrastriatal and Striatal Regions in Temporal Lobe Epilepsy
Source: PLoS One. 2015 Nov 6;10(11):e0141098. doi: 10.1371/journal.pone.0141098 (PMC4636381; doi:10.1371/journal.pone.0141098)
Supplement: S1 Table — 18F-Fallypride binding potential mean scores (± standard deviation) and statistics for regions of interest on epileptogenic and non-epileptogenic sides of all patients compared to controls. (DOC) [file pone.0141098.s005.doc]

Supplementary Table 1: ^18^F-Fallypride binding potential mean scores (± standard deviation) and statistics for regions of interest on epileptogenic and non-epileptogenic sides of all patients compared to controls

| **Region** | **Patients** | **Controls** | | **F** | | **df** | **p** |
| --- | --- | --- | --- | --- | --- | --- | --- |
|  |  |  |  | |  | |  |
| Temporal pole |  |  |  | |  | |  |
| - epileptogenic | 0.435 (± 0.146) | 0.794 (± 0.147) | 54.558 | | 1/36 | | 0.000 |
| - non-epileptogenic  Gyrus temp. sup. | 0.775 (± 0.156) | 0.761 (± 0.157) | 0.071 | | 1/36 | | 0.792 |
| - epileptogenic | 0.443 (± 0.153) | 0.589 (± 0.154) | 8.193 | | 1/36 | | 0.007 |
| - non-epileptogenic  Gyrus temp. sup. ant. | 0.579 (± 0.163) | 0.563 (± 0.164) | 0.086 | | 1/36 | | 0.771 |
| - epileptogenic | 0.482 (± 0.187) | 0.751 (± 0.188) | 18.899 | | 1/36 | | 0.000 |
| - non-epileptogenic  Gyrus temp. med. | 0.689 (± 0.195) | 0.689 (± 0.196) | 0.000 | | 1/36 | | 0.999 |
| - epileptogenic | 0.397 (± 0.158) | 0.519 (± 0.159) | 5.360 | | 1/36 | | 0.026 |
| - non-epileptogenic  Gyrus temp. med. ant. | 0.540 (± 0.152) | 0.535 (± 0.153) | 0.012 | | 1/36 | | 0.915 |
| - epileptogenic | 0.387 (± 0.159) | 0.544 (± 0.160) | 8.793 | | 1/36 | | 0.005 |
| - non-epileptogenic  Gyrus temp. inf. | 0.573 (± 0.152) | 0.568 (± 0.153) | 0.010 | | 1/36 | | 0.922 |
| - epileptogenic | 0.431 (± 0.178) | 0.644 (± 0.179) | 12.978 | | 1/36 | | 0.001 |
| - non-epileptogenic  Gyrus parahippocampalis | 0.652 (± 0.163) | 0.589 (± 0.164) | 1.369 | | 1/36 | | 0.250 |
| - epileptogenic | 0.584 (± 0.127) | 0.605 (± 0.128) | 0.252 | | 1/36 | | 0.619 |
| - non-epileptogenic  Hippocampus | 0.671 (± 0.136) | 0.594 (± 0.137) | 2.891 | | 1/36 | | 0.098 |
| - epileptogenic | 0.792 (± 0.168) | 0.768 (± 0.169) | 0.194 | | 1/36 | | 0.662 |
| - non-epileptogenic  Inferior parietal lobule | 0.769 (± 0.168) | 0.729 (± 0.168) | 0.511 | | 1/36 | | 0.479 |
| - epileptogenic | 0.382 (± 0.125) | 0.424 (± 0.126) | 1.007 | | 1/36 | | 0.322 |
| - non-epileptogenic  Midbrain | 0.438 (± 0.151) | 0.414 (± 0.152) | 0.228 | | 1/36 | | 0.636 |
| - epileptogenic | 1.156 (± 0.220) | 1.162 (± 0.221) | 0.007 | | 1/36 | | 0.936 |
| - non-epileptogenic  Caudate nucleus (head) | 1.164 (± 0.226) | 1.186 (± 0.227) | 0.084 | | 1/36 | | 0.774 |
| - epileptogenic | 14.86 (± 2.037) | 15.50 (± 2.047) | 0.896 | | 1/36 | | 0.350 |
| - non-epileptogenic  Putamen (ant.) | 15.21 (± 1.971) | 15.28 (± 1.981) | 0.010 | | 1/36 | | 0.923 |
| - epileptogenic | 17.48 (± 2.209) | 19.49 (± 2.220) | 7.491 | | 1/36 | | 0.010 |
| - non-epileptogenic  Putamen (post.) | 18.03 (± 2.046) | 18.80 (± 2.057) | 1.284 | | 1/36 | | 0.265 |
| - epileptogenic | 17.49 (± 2.230) | 19.02 (± 2.242) | 4.275 | | 1/36 | | 0.046 |
| - non-epileptogenic  Thalamus | 17.66 (± 2.181) | 19.63 (± 2.193) | 7.338 | | 1/36 | | 0.010 |
| - epileptogenic | 1.800 (± 0.311) | 1.857 (± 0.312) | 0.300 | | 1/36 | | 0.587 |
| - non-epileptogenic | 1.916 (± 0.321) | 1.742 (± 0.322) | 2.667 | | 1/36 | | 0.111 |
