# Supplementary material for: Reduced D2/D3 Receptor Binding of Extrastriatal and Striatal Regions in Temporal Lobe Epilepsy
Source: PLoS One. 2015 Nov 6;10(11):e0141098. doi: 10.1371/journal.pone.0141098 (PMC4636381; doi:10.1371/journal.pone.0141098)
Supplement: S2 Table — 18F-Fallypride binding potential mean scores (± standard deviation) and statistics for regions of interest on epileptogenic side compared to non-epileptogenic side of all patients. (DOC) [file pone.0141098.s006.doc]

Supplementary Table 2: ^18^F-Fallypride binding potential mean scores (± standard deviation) and statistics for regions of interest on epileptogenic side compared to non-epileptogenic side of all patients

| **Region** | **epileptogenic** | **non-epileptogenic** | **T** | **df** | **p** |
| --- | --- | --- | --- | --- | --- |
|  |  |  |  |  |  |
| Temporal pole | 0.542 (± 0.209) | 0.905 (± 0.294) | -6.941 | 1/15 | 0.000 |
| Gyrus temp. sup. | 0.560 (± 0.247) | 0.711 (± 0.289) | -3.516 | 1/15 | 0.003 |
| Gyrus temp. sup. ant. | 0.611 (± 0.253) | 0.847 (± 0.317) | 3.903 | 1/15 | 0.001 |
| Gyrus temp. med | 0.484 (± 0.236) | 0.654 (± 0.261) | -3.882 | 1/15 | 0.001 |
| Gyrus temp. med. ant. | 0.465 (± 0.223) | 0.679 (± 0.262) | -4.575 | 1/15 | 0.000 |
| Gyrus temp. inf. | 0.519 (± 0.243) | 0.742 (± 0.281) | -4.121 | 1/15 | 0.001 |
| Gyrus parahippocampalis | 0.682 (± 0.185) | 0.751 (± 0.212) | -1.769 | 1/15 | 0.097 |
| Hippocampus | 0.893 (± 0.198) | 0.867 (± 0.185) | 0.536 | 1/15 | 0.600 |
| Inferior parietal lobule | 0.479 (± 0.231) | 0.541 (± 0.269) | -1.948 | 1/15 | 0.070 |
| Midbrain | 1.298 (± 0.285) | 1.271 (± 0.220) | 0.765 | 1/15 | 0.456 |
| Caudate nucleus (head) | 15.74 (± 2.117) | 15.77 (± 1.890) | -0.130 | 1/15 | 0.899 |
| Putamen (ant.) | 18.51 (± 2.928) | 19.13 (± 2.870) | -1.774 | 1/15 | 0.096 |
| Putamen (post.) | 18.27 (± 1.668) | 18.66 (± 2.401) | -0.956 | 1/15 | 0.354 |
| Thalamus | 2.032 (± 0.461) | 2.060 (± 0.399) | -0.603 | 1/15 | 0.556 |
